# Online Tobacco Cessation Training and Competency Assessment for Complementary and Alternative Medicine (CAM) Practitioners: Protocol for the CAM Reach Web Study

**DOI:** 10.2196/resprot.5061

**Published:** 2016-01-06

**Authors:** Myra L Muramoto, Amy Howerter, Emery R Eaves, John R Hall, David B Buller, Judith S Gordon

**Affiliations:** ^1^ University of Arizona Department of Family & Community Medicine Tucson, AZ United States; ^2^ University of Arizona Biomedical Communications Tucson, AZ United States; ^3^ Klein Buendel, Inc. Golden, CO United States

**Keywords:** tobacco cessation, brief intervention, online training, communication, acupuncture, chiropractic, massage therapy

## Abstract

**Background:**

Complementary and alternative medicine (CAM) practitioners, such as chiropractors, acupuncturists, and massage therapists, are a growing presence in the US health care landscape and already provide health and wellness care to significant numbers of patients who use tobacco. For decades, conventional biomedical practitioners have received training to provide evidence-based tobacco cessation brief interventions (BIs) and referrals to cessation services as part of routine clinical care, whereas CAM practitioners have been largely overlooked for BI training. Web-based training has clear potential to meet large-scale training dissemination needs. However, despite the exploding use of Web-based training for health professionals, Web-based evaluation of clinical skills competency remains underdeveloped.

**Objective:**

In pursuit of a long-term goal of helping CAM practitioners integrate evidence-based practices from US Public Health Service Tobacco Dependence Treatment Guideline into routine clinical care, this pilot protocol aims to develop and test a Web-based tobacco cessation training program tailored for CAM practitioners.

**Methods:**

In preparation for a larger trial to examine the effect of training on CAM practitioner clinical practice behaviors around tobacco cessation, this developmental study will (1) adapt an existing in-person tobacco cessation BI training program that is specifically tailored for CAM therapists for delivery via the Internet; (2) develop a novel, Web-based tool to assess CAM practitioner competence in tobacco cessation BI skills, and conduct a pilot validation study comparing the competency assessment tool to live video role plays with a standardized patient; (3) pilot test the Web-based training with 120 CAM practitioners (40 acupuncturists, 40 chiropractors, 40 massage therapists) for usability, accessibility, acceptability, and effects on practitioner knowledge, self-efficacy, and competency with tobacco cessation; and (4) conduct qualitative and quantitative formative research on factors influencing practitioner tobacco cessation clinical behaviors (eg, practice environment, peer social influence, and insurance reimbursement).

**Results:**

Web-training and competency assessment tool development and study enrollment and training activities are complete (N=203 practitioners enrolled). Training completion rates were lower than expected (36.9%, 75/203), necessitating over enrollment to ensure a sufficient number of training completers. Follow-up data collection is in progress. Data analysis will begin immediately after data collection is complete.

**Conclusions:**

To realize CAM practitioners’ potential to promote tobacco cessation and use of evidence-based treatments, there is a need to know more about the facilitative and inhibitory factors influencing CAM practitioner tobacco intervention behaviors (eg, social influence and insurance reimbursement). Given marked differences between conventional and CAM practitioners, extant knowledge about factors influencing conventional practitioner adoption of tobacco cessation behaviors cannot be confidently extrapolated to CAM practitioners. The potential impact of this study is to expand tobacco cessation and health promotion infrastructure in a new group of health practitioners who can help combat the continuing epidemic of tobacco use.

## Introduction

Complementary and alternative medicine (CAM) practitioners such as chiropractors, acupuncturists, and massage therapists are a growing presence in the US health care landscape as a significant proportion of Americans report using CAM. The National Health Interview Survey (NHIS) periodically includes additional questions (Adult Alternative Medicine (ALT) supplement) on the use of CAM therapies. Clarke et al [1] examined trends in CAM use from the NHIS 2002, 2007, and 2012 ALT supplements, adjusting for methodological differences across the three surveys. They reported that the percentage of adults who had used any form of CAM in the preceding 12 months ranged from 32.3% in 2002 to 35.5% in 2007, and 33.2% in 2012. Among populations known to be at greater risk for tobacco use (eg, poor, lower educational attainment, public health insurance, and uninsured) there were a significant percentage using CAM in 2012. They found that 20.6% of poor adults, 24.4% of adults with a high school diploma or GED, 24.8% of public insurance, and 22.9% of uninsured adults reported using CAM [1]. An analysis of 2007 NHIS data found that significant numbers of respondents who used CAM services in the prior year had the following chronic disease and/or major risk factors: (1) current smoking (17.4%), (2) hypertension (18.1%), (3) obesity (21.4%), and (4) physical inactivity (22%) [2].

Tobacco use accounts for nearly 443,000 deaths in the United States each year and is responsible for US $96 billion in health care expenditures annually [3], yet use of effective cessation aids remains low. Of the 45.3 million tobacco users in the United States in 2010 [4], 52.4% reported a quit attempt in the previous year [5,6]. Overall, nearly 69% of smokers report they want to quit [7]. Effective tobacco cessation treatments recommended by the US Public Health Service Guideline on Treatment of Tobacco Dependence (PHS Guideline) [8], are more widely available than ever, and yet are still greatly underutilized. Nearly 70% of tobacco users attempt to quit without assistance [7] and unaided attempts are rarely successful [9]. There is an urgent need to increase use of proven cessation aids.

Decades of public health tobacco control efforts have led to steady declines in tobacco use prevalence from 40.3% in 1964 to 19.3% in 2011, and 19.2% in 2013 [4,10,11]. But in recent years inconsistency or stagnation in this downward trend has prompted public health calls for new and expanded strategies for approaching tobacco cessation, including broadened insurance coverage for cessation treatment [5]. CAM practitioners have the opportunity to intervene with chronic disease risk factors such as tobacco use, and in at least one study, chiropractors were found to be more likely to engage in tobacco cessation activities with patients than primary care physicians [12].

With a few exceptions [13-16], CAM practitioners have been largely overlooked in the nation’s tobacco control agenda. For more than two decades, public health efforts have targeted physicians for tobacco brief intervention (BI) training [17]. Only more recently has BI training been offered to other biomedical health care professionals (nurses, dentists, pharmacists) [17]. Despite clear evidence that asking, assessing, and intervening by health care providers results in increased quit rates [8], biomedical physicians do not consistently offer tobacco BIs. An Association of American Medical Colleges survey found that 86% of physicians advise patients to stop tobacco use but only 37% discuss counseling options, 31% recommend nicotine replacement, and 13% refer patients to others for cessation treatment [18]. There is a clear need to expand research on tobacco cessation training beyond conventional health practitioners to increase the potential public health reach and impact of brief tobacco cessation interventions.

Web-based training has clear potential to meet large-scale training dissemination needs. Yet, despite widespread use of Web-based instruction for training health professionals [19], few online tools are available for assessing clinical skills competency**.** While initial development costs are higher for online training, costs for ongoing training delivery are low. Compared with classroom instruction, online training offers additional advantages including greater learner accessibility, increased convenience, and greater scalability. However, online evaluation tools for assessing clinical skills competency with similar accessibility, scalability, and low dissemination costs are still lacking. In preparation for a larger validation study, this study aims to create an online, scalable tool to assess clinical skills competency among CAM practitioners and to assess the feasibility of comparing this tool’s performance to a live video standardized patient method of learner assessment.

Innovative aims of this study include (1) shift the current public health and clinical practice paradigm in tobacco cessation training, currently focused on conventional biomedical practitioners, to include training CAM practitioners in evidence-based knowledge and skills to promote tobacco cessation and encourage use of effective treatments; (2) develop a unique, Web-based tobacco cessation BI training program for CAM practitioners to improve their competency in helping their clients or patients quit tobacco; (3) develop a new Web-based tool to assess CAM practitioners’ clinical skills competency at providing tobacco BIs; and (4) fill a gap in the literature by collecting new information on CAM providers regarding online BI training, clinical competency testing, and the facilitating and inhibiting role of practice environment, insurance reimbursement, and training availability on adoption of tobacco cessation BI behaviors. Initial components of the study that have already been completed are described in the Results section. We note that the three CAM disciplines participating in the CAM Reach Web (CAMR-Web) study customarily use different terms to refer to persons seeking their care. Chiropractors and acupuncturists usually refer to “patients”, whereas massage therapists usually say “clients”. For simplicity, we will use “patients” throughout this paper.

## Methods

The CAMR-Web study is a mixed-method (qualitative and quantitative) pilot feasibility study. Theoretical frameworks informing this work are Social Cognitive Theory [20], Adult Learning Theory [21,22], and Swing’s [23] competency-based education and assessment framework, which informs the competency assessment tool development. It builds upon the BI curriculum and training development work completed in our prior CAM Reach (CAMR) study (NCI 1RO1 CA137375) [15,16]. The CAMR-Web study has three overlapping phases: Phase 1 (development of training website and competency assessment tool (completed)), Phase 2 (training website pilot study and assessment tool pilot validation study (underway), and Phase 3 (data synthesis and write-up (planned)).

CAM practitioners differ from conventional practitioners in substantive ways such as professional training, scope of practice, philosophies of practice and approaches to healing; practice patterns, and patient or client relationships [12,24-26]. Accordingly, the numerous tobacco cessation-training programs available to conventional practitioners are not well suited for CAM practitioners. In our previous CAMR study [15,16,27,28], we developed an in-person training workshop and practice system intervention that is uniquely and specifically tailored for CAM practitioners. It is based on extensive formative research with expert review and input from leaders in CAM education and research, tobacco cessation interventions and policy, and integrative medicine [16]. The CAMR study results showed that CAM practitioners had significant increases in tobacco cessation activities, motivation and confidence in helping patients quit tobacco, and comfort with providing information and referrals for PHS Guideline-based tobacco cessation aids. These increases occurred across all three practitioner types and were sustained at 12 months, despite heterogeneity in professional training, practice patterns and organization, and practice business models. Adapting the CAMR training for Web-based delivery was intended to create a unique tobacco BI training that is more accessible and scalable.

Despite growth of Web-based training of health care practitioners, effective Web-based methodologies to assess clinical competencies lag behind. “Standardized patient” is an evaluation method widely used in medical education for assessing clinical skills competency [29]. It most closely simulates a real patient encounter and is thus a major component of high-stakes exams (eg, Objective Structured Clinical Examination (OSCE) required for certification and licensure) [30,31]. Use of OSCEs and standardized patients in CAM practitioner education and evaluation is relatively new and uncommon; however, there is a gap in the literature on the use of these methodologies in CAM education and competency assessment. This study was designed to fill a need for innovative, scalable, Web-based tools to evaluate clinical skills competency that can approximate simulation of in-person methods such as a standardized patient.

### Phase 1 (Complete)

Phase 1 of the study (completed) involved evaluation of the original CAMR study training curriculum for the adaptation necessary for Web-based training delivery of the content, followed by design and production of the training website. Specific methods for accomplishing these aspects are detailed in the following sections.

#### Design and Production of Training Website

The curriculum and Web development teams worked collaboratively in the creation of the CAMR-Web training content map and specifications to best suit the curriculum. This team of investigators and staff had extensive experience working together to develop online tobacco cessation trainings. The content map and specifications served as a guide for the design process which began with storyboarding interactive learning activities and course content, followed by developing “screenplay” versions of learning activities and content and identifying needed animation or video assets and/or interactive simulations to reinforce and demonstrate desired instructional objectives within the content map. Database connectivity was designed to facilitate (1) the collection of demographic data from users in a convenient, easy-to-analyze format; (2) the use of open- and closed-ended evaluation measures; (3) the collection and instantaneous scoring of nominal and ordinal evaluation data; (4) the ability to track user progress through curriculum modules and the site as a whole; and (5) the ability to deploy dynamic, customized Web pages created in real-time for each user.

#### Adaptation of CAMR Training Content for Online Delivery

Investigators adapted the existing CAMR in-person, multimedia training to best suit the online learning environment. Continuing the highly successful, inclusive approach to developing the CAMR in-person curriculum, adaptation of the curriculum for Web-based delivery was conducted in consultation with external reviewers from our local and national advisory panels. Upon project startup, local advisors were invited from our pool of completed CAMR study participants. In tandem with this curriculum content conversion, we developed customized clinical cases to assess core competencies addressed by the CAMR Web online curriculum. We then deployed the clinical cases through the DecisionSim simulation development learning platform [32], integrating the clinical cases in such a way as to provide for incremental and summative learner competency assessment as described below.

#### Adaptation of CAMR Instruments and Measures for Online Data Collection

Evaluation instruments including screening, baseline, pretraining and post-training assessments, and follow-up surveys were developed or adapted from existing instruments developed for the CAMR study [16,27,33]. They were pilot tested with individuals from the target audiences and reviewed by our advisory panels. User interface and database back-end components were constructed once instruments were finalized.

### Development and Production of Simulation Cases and a Web-Based Competency Assessment Tool

Using DecisionSim, the summative assessment is an online simulation of a case-based practice of communication skills and application of tobacco cessation knowledge. The learner is presented with a tobacco-using patient or client scenario for which an “ideal” interaction path has been previously specified by investigators, in consultation with our advisors. Each response option is associated with a tag (in the back-end database) as being “optimal”, “critical”, or “poor.” To successfully complete the training a learner needs to meet a minimum level of competence as defined by selection of a response path that is within a specified range of the “ideal” interaction path. To evaluate the online competency tool, we also developed a standardized patient “live simulation” role play exercise in which participants role play the exercise scenarios with a standardized patient via Skype video. Live simulation cases reflect a range of patients’ readiness to quit and evaluate the practitioner’s ability to assess the patient or client and adjust their BI behaviors accordingly. Standardized patient cases, scripts, and examiner checklists for the feasibility study were developed in tandem with simulation cases for the competency assessment tool. For both the live and online simulation cases, we developed related evaluation criteria and thresholds for determining competency. Development of evaluation criteria and thresholds were guided by published, evidence-based tobacco cessation behavioral support competencies and guidance documents (eg, those supported by national tobacco cessation programs [34]) and review and feedback from our advisory panels.

### User, Usability, and Beta Testing of the CAMR Website and Competency Assessment Tool

The CAMR website underwent rigorous user and usability testing with local and national panel members as well as with members of the target population of CAM practitioners. Investigators reviewed the initial version of the training program and made modifications to improve usability. The initial version was also thoroughly tested in-house for code errors. It was tested for usability with volunteer CAM practitioners (N=18; 6 chiropractors, 3 massage therapists, 9 acupuncturist) with protocol analysis. Practitioners were given a set of tasks to mimic real-world use (eg, registering on the site, viewing the training, completing the competency assessment). Testers completed a postsurvey on ease of use and attractiveness of screen design [35]. Investigators and programmers reviewed the results and made changes to improve usability.

Near completion, the CAMR website was beta tested with CAM practitioners and advisory panel members (N=12). The goal of beta testing was to confirm that revisions made in response to usability study findings eliminated identified problems and that all site features function as intended. Beta testing also gauged the instructional effectiveness of the curriculum. The process followed protocols suggested by Trollip and Alessi [36], which included (1) beta testers have content expertise matching the expected, pre-existing knowledge base of the prototypical learner; (2) content quizzes employing the post-test instruments are used to conduct item analysis of online quizzes and learning activities and to gauge the effectiveness of the Web-based curriculum; and (3) testers are interviewed to identify attitudes toward the instructional experience.

### Phase 2 (Underway)

#### Feasibility Study of CAMR-Web-Based Training

The feasibility study design is a single group with participant assessments at (1) baseline (study enrollment) and pretest prior to CAMR-Web training intervention; (2) immediately post-training; and (3) 3 and 6 months post training completion. A subsample of practitioners from each practitioner type was selected based on specific criteria to complete an in-depth, open-ended response follow-up survey on selected factors influencing practitioner adoption of tobacco intervention behaviors (eg, practice environment, peer social influence, and insurance reimbursement). Phase 2 participant enrollment is complete and data collection is still in progress.

#### Participant Recruitment, Eligibility, and Enrollment

Based on literature regarding attrition among online learners [37,38], and our own experience delivering online training to other target populations [39,40], we anticipated that roughly 50.0% (60/120) of participants would complete all parts of the proposed study activities. Given this estimate, we originally planned to recruit a total of 120 practitioners (40 per CAM discipline), with the goal of retaining at least 19 practitioners from each discipline through the end of the study. We recruited practitioners through websites, listservs, and newsletters of CAM practitioner professional organizations, alumni of schools of chiropractic, acupuncture, and massage therapy, and through the national provider network of a large third-party payer for chiropractic, acupuncture, and massage therapy services. Interested practitioners were directed to the project website for information about the study and access to screening and enrollment instruments.

To participate, practitioners must be ≥18 years of age, have unrestricted license or credentials to practice their CAM discipline, be actively in practice three quarters time to full-time, see at least 10 patients per week, have access to a computer with broadband Internet access and audio output, and be willing to provide consent and participate in the entire study. Practitioners were excluded if they reported participation in formal tobacco cessation training in the past 2 years.

Practitioners arriving at the project website were presented with a project overview. To enroll, practitioners first completed the screening instrument to determine eligibility. Responses to the screening items were processed immediately and the eligibility status displayed to the practitioner. Eligible participants were directed to a link to the consent portal. This portal displayed the consent document in its entirety with a link to download and print the form. To continue with enrollment, the participant was required to click a button indicating that he or she has read and agrees to all parts of the online consent form. Next, the online baseline and pretraining survey were presented for completion. (Textbox 1). Once completed, participants were enrolled and provided full access to the CAMR-Web training.

#### Participant Training (Completed) and Follow-Up (in Progress)

Enrolled practitioners experience the CAMR-Web tobacco cessation BI curriculum [16], which was tailored for CAM practitioners and focuses on a nonconfrontational approach that is referred to as a “helping conversation.” In our experience developing the in-person CAMR curriculum [16], transforming the “5 A’s” protocol [8] into a less proscriptive and more motivational framework is more acceptable to these CAM practitioner groups. Practitioners are encouraged to complete the CAMR-Web training within 4 weeks. Upon finishing the training, practitioners are asked to complete the online competency assessment tool and post-training assessments. A training certificate is issued to all practitioners who successfully demonstrate the requisite competency level and who respond correctly to at least 80% of knowledge items. Practitioners receive compensation for completing the research assessment and eight continuing education credits for completing the online training. Practitioners also receive a training completion packet by mail with twenty copies of each patient handout. They may request additional copies as needed throughout their study participation.

After training, practitioners are asked to complete Web-based questionnaires at 3 and 6 months post training completion (Textbox 1). These follow-up intervals are based on our prior work with community-based tobacco cessation training of nonpractitioners and on the current CAMR study. Follow-up in less than 3 months does not allow sufficient time for participants to have implemented their training, while changes in self-efficacy measures tend to stabilize by 6 months. Participants receive up to US $110 incentive for completion of all study assessments.

**Summary of CAMR evaluation instruments, measures, and domains.**

Measure and addressed domains

- Screening
  - Eligibility criteria
- Baseline and pretest assessment
  - Demographics
  - Cessation intervention behaviors, attitudes regarding tobacco use, quitting, and intervening with patients
  - Confidence and self-efficacy in performing intervention behaviors
  - Training-related knowledge [27]
- Post-training assessment
  - Cessation intervention behaviors, attitudes regarding tobacco use, quitting and intervening with patients,
  - Confidence and self-efficacy in performing intervention behaviors,
  - Training-related knowledge,
  - Training site usability [35].
- Web-based skills competency assessment
  - Ability to recognize and select appropriate: (1) Techniques to ask or become aware of patient or client tobacco use; (2) Communication skills to assess tobacco use of client/patient; (3) Advice and encouragement to consider quitting tobacco; (4) Assistance (eg, referral to guideline based services and provision of materials or information); (5) Arrangements to follow up with patient or client regarding tobacco use.

- Follow-up assessments at 3 and 6 months
  - Cessation intervention behavior and use of training materials,
  - Confidence and self-efficacy in performing intervention behaviors
- In-depth, open-ended follow-up survey

- Open-ended, in-depth survey with a subsample of participants

### Competency Assessment Tool Validation Feasibility Pilot

This feasibility pilot study is using the University of Arizona College of Medicine’s standard protocols for training standardized patients. To test feasibility for a larger validation study, we recruited a subsample of 10 CAM practitioners who completed the CAMR-Web training (n=3-5 per practitioner type) to participate in the live video standardized patient interviews to assess CAM practitioner skill competency. Eligible participants must have completed training within the prior 2 weeks and have access to bi-directional Internet video capability (eg, Skype) sufficient to participate in a 30-minute, live video, standardized patient role-play session. Each participant is evaluated with two standardized patient cases (1 ready to quit, 1 not ready to quit), and each case is scored separately. Practitioners are not offered feedback on their performance to minimize the impact of the standardized patient on practitioner behavior during the remainder of the follow-up period. Practitioners who complete the role-play are then invited to complete a survey about usefulness of the activity. Their live assessment scores are then compared to their online competency assessment scores.

### In-Depth, Open-Ended Response Survey of Practitioner Subsample

Approximately 6 months after training completion, practitioners are screened for eligibility to complete an in-depth online survey with open-ended response options. Inclusion criteria into this survey require that (during their 3-month follow-up survey) practitioners report seeing tobacco users in their practice and respond with “always” or “often” on a 4-point scale (always, often, sometimes, never) to at least 3 of the following (1) assessing interest in quitting; (2) identifying reasons to quit; (3) offering materials; (4) discussing medications; (5) treating tobacco addiction; or (6) referring to tobacco treatment outside of their practice.

Open-ended responses from the online survey will be analyzed using qualitative methodology to gain a more detailed understanding of practitioners’ experiences as they apply training in real-world settings [41]. Open-ended survey questions were designed to gather formative data on specific factors that might influence CAM practitioner adoption of tobacco BI behaviors to inform a future dissemination trial. Investigators used interview guides from the CAMR study to design items focused on practice environment, peer social influence, and third-party reimbursement. Question themes focused on (1) facilitating or inhibiting factors in practice environment; (2) desirability and utility of peer social networking; and (3) reimbursement issues such as eligibility, qualifications and credentialing, documentation, and price-point. Reimbursement themes will be explored only at the end of the study to minimize potential effects on practitioner behavior during the post-training observation period.

### Sample Size

Data for power analysis came from pretest and post-test assessments of knowledge and confidence (self-efficacy) in the previous in-person CAMR training study. We compared the number of correct answers on a 15-item tobacco cessation knowledge test administered before and after training for 33 practitioners. Scores on the items approximated a normal distribution. A paired *t* test analysis indicated significant improvement in knowledge (*t*=6.7, *P*<.001). Using the data from the 33 practitioners (mean (SD) of the paired differences is 2.1(1.8)), a *t* test power analysis indicated that 10 subjects would be needed for power =.90 and alpha =.05 and 11 subjects needed for the Wilcoxon test which makes fewer assumptions about the distribution of the scores than does the *t* test and typically requires a larger sample.

The pretest and post-test confidence question was “I am confident that I can personalize the benefits of quitting with each individual patient”. The possible responses ranged from 1 (“Not at all confident”) to 4 (“Very confident”). The scores on this question approximated a normal distribution. A paired *t* test analysis indicated a significant positive change (*t*=5.9, *P*<.001). Using the data from the 33 practitioners (mean (SD) of the paired differences is 0.7(0.84)), a *t* test power analysis indicated that 18 subjects would be needed for power = .90 and alpha = .05. For the Wilcoxon test, 19 subjects would be needed. Power analyses were conducted using PASS (Version 11.0.8) from NCSS Statistical Software.

### Phase 3 (Planned)

The process of using the CAMR-Web training website is being evaluated by assigning CAM providers IDs, which mark usage in the backend database, including time spent, pages viewed, and modules completed. The association of use of the website with change in knowledge and confidence will be assessed using regression analysis methods. These analyses will be limited by use not being randomly assigned so potential third variables (eg, demographics, years in practice, patient volume) will be included as covariates.

In so far as knowledge and confidence were measured at baseline, 3, and 6 months (for three practitioner types), changes will be analyzed using mixed-models [42]. Adjustments will be made for participant characteristics (eg, demographics, years in practice, patient volume, and provider group for each CAM group). This approach will enable us to simultaneously assess intervention effects for participants overall and by group. The analysis will be conducted using SAS 9.4 [43]. Intra-class correlation coefficients will be calculated between CAM providers’ knowledge and confidence measures, and clinical skills scores after completing the new DecisionSim Web-based CAMR competency tool and the live standardized patient assessment. Positive correlations at *P*<.05 were planned to establish predictive validity.

Qualitative analysis of open-ended survey responses will use a coding-categorizing technique [44,45]. This qualitative strategy is a form of content analysis that involves arranging the data into categories sorted by broader themes (eg, patient presenting symptom, events, etc) to assess issues not easily captured by closed survey questions and to generate themes for further study.

## Results

We have enrolled 203 practitioners (63 chiropractors, 78 acupuncturists and traditional Chinese medicine practitioners, and 62 massage therapists). Initial training completion rates varied by CAM discipline, but overall the average completion rate was lower (36.9%, 75/203) than we expected based on our prior experience with other Web-based training. We therefore overenrolled to meet the original target of 20 practitioners per CAM group completing training and 3-month follow-up. At the time of writing, all training activities have been completed and follow-up survey data collection is in progress. Final data collection will be completed in November 2015. In Phase 3, we will analyze the main outcomes, synthesize results from quantitative and qualitative data, evaluate the feasibility of a larger competency assessment tool validation study, and address the feasibility outcomes in preparation for a larger dissemination study.

## Discussion

### Principal Findings

Focusing tobacco BI training on conventional practitioners limits BIs’ potential public health impact. Unhealthy lifestyle behaviors are the root cause of the growing burden of chronic disease in the United States [46-49]. Tobacco use, diet, and physical activity are three lifestyle behaviors affecting the nation’s public health that are major risk factors for the most prevalent chronic diseases: cardiovascular disease, diabetes, and cancer. The Institute of Medicine report on CAM use in the United States found little research on the role of CAM in addressing national public health priorities requiring behavioral change [50]. This has also been recognized by the naturopathic physician and chiropractic communities [2]. Key questions remain regarding (1) the role of CAM practitioners in fostering and sustaining behavior change around tobacco use and other health behaviors; (2) CAM practitioners’ behaviors related to promoting healthy behavior; (3) patients’ use of CAM practitioners to support behavior change; (4) the potential role of CAM practitioners in preventive and promotive health; and finally (5) whether CAM practitioners can be engaged to fully participate in a public health community of practice [51]. Tobacco cessation can serve as a model with which to examine these issues.

Factors impacting CAM practitioner tobacco cessation behaviors are not well studied, especially regarding adoption of PHS Guideline-based tobacco dependence treatment practices. Among conventional practitioners, training [17], practice environment system changes [52-54], and pro-adoption peer social influences have generally been shown to increase tobacco BI behaviors, particularly in combination with one another [55]. Prior to this study, however, there was little to no research on how these factors affect CAM practitioners. Effects of insurance reimbursement on conventional practitioner tobacco BI behavior are mixed [56,57], and in the case of Medicare reimbursement evidence suggests underutilization of billing for cessation counseling services [58]. Insurance reimbursement for tobacco cessation services is now widely available for conventional medical practitioners, although not presently for CAM practitioners. Given this, and other substantive differences, extant knowledge about factors promoting conventional practitioners’ adoption of tobacco intervention behaviors cannot be confidently extrapolated to CAM practitioners.

### Conclusions

CAM practitioners are an increasingly important presence in the US health care system and already provide health and wellness care to significant numbers of patients who use tobacco and/or have other chronic disease(s) and risk factors. There has been scant research, however, into the potential factors impacting on CAM practitioners’ willingness and ability to adopt evidence-based tobacco cessation behaviors as part of routine clinical practice. These factors include cessation training adapted to their professional discipline, professional scope of practice, and approaches to health and wellness. Other potential factors include practice organization and business models, and the availability of third party insurance reimbursement for tobacco cessation services. The keys to insurance reimbursement are proven competency and credentials, and evidence-based deployment of an effective intervention. To engage CAM practitioners in tobacco cessation, it is critical to know how best to increase incorporation of evidence-based interventions and practices into their routine clinical care. Similarly, research on the effect of insurance reimbursement is critical to develop evidence-based policy for CAM practitioners to provide effective cessation interventions. The potential impact of this study is to build infrastructure in a new group of health practitioners who can help combat the continuing epidemic of tobacco use.

## Abbreviations

ALT: Adult Alternative Medicine
BI: brief interventions
CAM: chiropractors, acupuncturists, and massage
CAMR: CAM Reach
CAMR-Web: CAM Reach Web
NHIS: National Health Interview Survey
OSCE: Objective Structured Clinical Examination
PHS: Public Health Service
